# The development, incidence and treatment trends of trochanteric fractures in Germany: a cohort study

**DOI:** 10.1186/s13018-023-03981-5

**Published:** 2023-07-10

**Authors:** Yannick Rau, Jasper Amtsfeld, Nils Reimers, Ludwig Matrisch, Jasper Frese, Arndt-Peter Schulz

**Affiliations:** 1 Faculty of Medicine, Universität zu Lübeck, Lübeck, Germany; 2 Zentrum Klinische Forschung, BG Klinikum Hamburg, Hamburg, Germany; 3 Chair of Technology Management, Christian-Albrechts-Universität zu Kiel, Kiel, Germany; 4 Stryker Trauma GmbH, Schoenkirchen, Germany; 5 Queensland University of Technology, Brisbane City, Australia; 6 Department of Trauma Surgery, Orthopaedics and Sports Traumatology, BG Klinikum Hamburg, Hamburg, Germany

**Keywords:** Trochanteric fractures, Trauma, Osteosynthesis, Epidemiology, Incidence

## Abstract

**Background:**

Hip fractures are a major public health problem worldwide and can lead to disability, increased mortality, and reduced quality of life. We aim to provide a nationwide epidemiological analysis of trochanteric and subtrochanteric fractures and their respective surgical treatments.

**Methods:**

Data were retrieved from the national database of the German Department of the Interior. ICD-10-GM and OPS data from the period of 2006 to 2020 were analysed and all patients with trochanteric and subtrochanteric fractures as their main diagnosis, who were treated in a German hospital, were included. Patients were grouped by age and gender and linear regression was performed where suitable to calculate statistically significant correlations between variables and incidences.

**Results:**

985,104 pertrochanteric fractures and 178,810 subtrochanteric fractures were reported during the analysed period. We calculated a mean incidence of 80.08 ± 6.34 for pertrochanteric and 14.53 ± 1.50 for subtrochanteric fractures per million inhabitants. In both fracture types, a distinct dependence of incidence on age can be determined. Incidence rates equally rise in both sexes through the age groups with an increase of about 288-fold from those under the age of 60 to those over the age of 90 in pertrochanteric fractures, and about 123-fold in subtrochanteric fractures. Intramedullary nailing was the most common kind of treatment for both fracture types with augmentative cerclages on the rise throughout the whole period. Plate and dynamic compression screws were decreasing in frequency over the analysed period in both fractures.

**Conclusions:**

We provided incidence data on per- and subtrochanteric fractures and their treatment. We calculated an economic impact of approximately 1.563 billion € per year in Germany. With regards to recent literature on costs of treatment and our findings regarding the implementation and utilization of different treatment methods, we conclude that the reinforcement of nationwide prevention programs is a relevant step in lessening the economic burden. We welcome the increased utilisation of intramedullary nailing as many studies show beneficiary outcomes and cost effectiveness in most of the included fracture types.

## Introduction

Hip fractures, with a global age standardised incidence rate (per 100,000 population) of 187.2 (2019), are a major public health problem worldwide and can lead to disability, increased mortality, and reduced quality of life [1–4]. With a 7.71 billion population worldwide in 2019, hip fractures, in general, are affecting around 14.43 million people per year globally [5].

They are a heterogeneous group with two main types of fractures: the extracapsular (trochanteric and subtrochanteric) fractures and the intracapsular (cervical or femoral neck) fractures. Most studies reporting about the epidemiology of fractures in the hip area summarize all types of injuries, as their epidemiology is very similar [6–10]. If reported, the distribution between these varies slightly between populations but are overall evenly distributed [11–16].

The vast majority of trochanteric and subtrochanteric hip fracture patients are elderly fragile patients with decreased bone quality, a tendency to fall, as well as an increased risk of major morbidity and mortality [13, 17–19]. In a study examining all fall-related fractures in a monocentric in- and outpatient population, proximal femoral fractures had the second highest prevalence in all fall-related fractures, the highest prevalence in all fall-related fractures above the age of 59 years, and the highest percentage of fall-related fracture type with 93.4% [8].

The influence of osteoporosis on the development of hip fractures has been well described, it has been calculated that with a worldwide prevalence of 19.37% in men and 51.48% in women over the age of 79, at least 50% of hip fractures occur in relation to osteoporosis [19–23].

Trochanteric fractures are commonly treated using a dynamic hip screw (DHS) or an intramedullary nail (IMN) [24, 25].

Current data clearly show an increased morbidity and mortality in clinical studies comparing surgical to non-surgical management [18, 26].

In 2019, Rupp et al. reported trochanteric fractures as the second most common type of fracture requiring hospital treatment in Germany with an incidence of 108.7 per 100,000 inhabitants. As trochanteric fractures together with fractures of the neck of the femur have a very high socioeconomic impact, large efforts have been made in the last 20 years to try and reduce the risk for sustaining a hip fracture in Germany. This includes the treatment of osteoporosis, programs and training modules for fall prophylaxis to improve strength and coordination, hip protector devices, and home visit programs to minimise domestic hazards [23, 27–31]. Although most of these interventions have been researched in the past, no data regarding an overall impact of these developments on the overall incidence of trochanteric fractures has been published so far. Population-based models of the potential impact of fall prevention exercise and oral bisphosphonates showed that very high treatment and participation rates are needed to achieve substantial effects on the occurrence of proximal femoral fractures [32].

In this study, we provide and elaborate on the epidemiology of trochanteric and subtrochanteric fractures, as well as their most common kinds of osteosynthesis, on a nationwide scale in Germany. We then aim to discuss the economic and public healthcare implications of the matter.

## Methods

The data used in this study were provided by the German Federal Bureau of Statistics, Department of the Interior (DESTATIS). Hospitals treating patients insured by statutory health insurance in Germany are required to report the main treatment diagnosis using the ICD-10-GM codes in the version published by the German Federal Institute for Drugs and Medical Devices (BfArM) and procedural details categorised in OPS-codes, the German equivalent to the WHO ICPM (International Classification of Procedures in Medicine) codes, also published by BfArM. The numbers are subdivided by patient sex and age in 5-year intervals within the national database.

For this study, we identified all patients from 2006 to 2020 who were coded by discharge from a hospital with an ICD-10-GM code S72.1 for pertrochanteric femoral fracture or S72.2 for subtrochanteric femoral fracture.

Patients under the age of 60 were summarised and patients over 60 were categorised in 10-year intervals up to 90 years old and older. The osteosynthesis methods considered in this study were all IMN techniques of proximal femur fractures and extramedullary fixation using DHS or plates.

The corresponding OPS codes were identified as 5–790.XX (closed reposition), 5–791.XX (open reposition of simple fractures of the shaft), 5–792.XX (open reposition of fragmented fractures of the shaft), 5–793.XX (open reposition of simple fractures in the joint region), and 5–794.XX (open reposition of fragmented fractures in the joint region). The suffix.XX is a placeholder for further distinction as the first letter indicates the treatment method used and the second letter indicates the anatomic location. For example, OPS 5–790.8f represents a closed reposition of a fracture with a dynamic compression screw (0.8) of the proximal femur (f). An overview of all analysed OPS Codes is shown in Table 1.

**Table 1:** Analyzed OPS codes, sorted by category

Closed reduction	Open simple reduction of the shaft	Open multiple fragment reduction of the shaft	Open simple reduction in the joint region	Open multiple fragment reduction in the joint region
5–790.0f	5–791.0g	5–792.0g	5–793.0f	5–794.0f
5–790.0g	5–791.1g	5–792.1g	5–793.1f	5–794.1f
5–790.1f	5–791.2g	5–792.2g	5–793.2f	5–794.2f
5–790.1g	5–791.3g	5–792.3g	5–793.3f	5–794.3f
5–790.2f	5–791.4g	5–792.4g	5–793.4f	5–794.4f
5–790.2g	5–791.5g	5–792.5g	5–793.5f	5–794.5f
5–790.3f	5–791.6g	5–792.6g	5–793.8f	5–794.7f
5–790.3g	5–791.7g	5–792.7g	5–793.af	5–794.8f
5–790.4f	5–791.9g	5–792.8g	5–793.bf	5–794.af
5–790.4g	5–791.gg	5–792.9g	5–793.cf	5–794.bf
5–790.5f	5–791.kg	5–792.kg	5–793.kf	5–794.cf
5–790.5g	5–791.xg			5–794.gf
5–790.6f				5–794.kf
5–790.6g				5–794.xf
5–790.7f				
5–790.8f				
5–790.9f				
5–790.nf				
5–790.xf				

In our analysis of treatment procedures, we opted to analyse the development of nailing and plating, in which we also included the dynamic compression or hip screw in both fractures, as they are the most common among surgical treatments now. Other options, like the fixateur externe, screws, or primary endoprosthesis, were also deployed and reported but are not specifically analysed here. Conservative approaches are not categorised within the OPS system and can therefore not be quantified in this study.

In addition to nailing and plating approaches, augmentative cerclages are also analysed and quantified with respect to their implementation in fracture treatments.

Adjustments for population growth (incidence rates) were calculated based on annualised population counts for Germany provided by DESTATIS.

All calculations and statistical analysis were performed using Microsoft Excel (Version 2207, Microsoft, Redmond, USA) and the R based Software Solution Jamovi (Version 2.2.5, The Jamovi Project, Sydney, Australia). For linear regression and student t-test analysis, an alpha error probability of *p* = 0.05 was assumed. Nagelkerke’s adjusted *R*^2^ and *F* test results are provided for regression models to quantify model fitness. Distribution assumptions of standardised residuals were checked via QQ plot analysis, and in graphically uncertain distributions with the Shapiro–Wilk test prior to performing regression.

## Results

Overall incidence of each diagnosis was calculated by using the ICD-GM codes and population size at the year of report. Overall, 985,104 pertrochanteric fractures and 178,810 subtrochanteric fractures were reported. Detailed age and sex adjusted mean incidences over the study period are provided in Table 2.

**Table 2:** Incidences of subtrochanteric and pertrochanteric fractures

Diagnosis	Sex	Age	Mean incidence per 100,000 ± standard deviation
Pertrochanteric fractures	Combined	All ages	80.08 ± 6.34
		< 60	6.46 ± 0.32
		61–70	51.71 ± 2.97
		71–80	175.52 ± 9.79
		81–90	770.67 ± 50.43
		> 90	1871.54 ± 144.33
	Male	All ages	46.38 ± 6.08
		< 60	8.58 ± 0.53
		61–70	52.62 ± 3.08
		71–80	132.94 ± 9.90
		81–90	476.32 ± 24.54
		> 90	1215.16 ± 218.27
	Female	All ages	112.61 ± 6.83
		< 60	4.27 ± 0.15
		61–70	50.85 ± 3.20
		71–80	210.42 ± 12.64
		81–90	933.57 ± 59.24
		> 90	2082.37 ± 133.57
Subtrochanteric fractures	Combined	All ages	14.53 ± 1.50
		< 60	2.15 ± 0.12
		61–70	11.64 ± 0.73
		71–80	34.32 ± 2.53
		81–90	126.06 ± 5.55
		> 90	265.37 ± 32.58
	Male	all ages	9.01 ± 1.14
		< 60	2.86 ± 0.25
		61–70	11.12 ± 0.85
		71–80	24.87 ± 2.52
		81–90	74.25 ± 6.53
		> 90	159.35 ± 41.89
	Female	all ages	19.86 ± 1.89
		< 60	1.41 ± 0.06
		61–70	12.12 ± 0.78
		71–80	42.07 ± 2.82
		81–90	154.98 ± 7.95
		> 90	299.31 ± 31.61

In both fracture types, a distinct dependence of incidence on age can be determined. In both sexes, incidence rates rise through the age groups with an increase of about 288-fold from those under the age of 60 to those over the age of 90 in pertrochanteric fractures and about 123-fold in subtrochanteric fractures.

While in both fracture types under the age of 60, male incidence is about two times that of female incidence, this observation almost completely inverts in the older age groups. A significant development of this dependency on sex over the study period could not be shown.

**Fig. 1:**
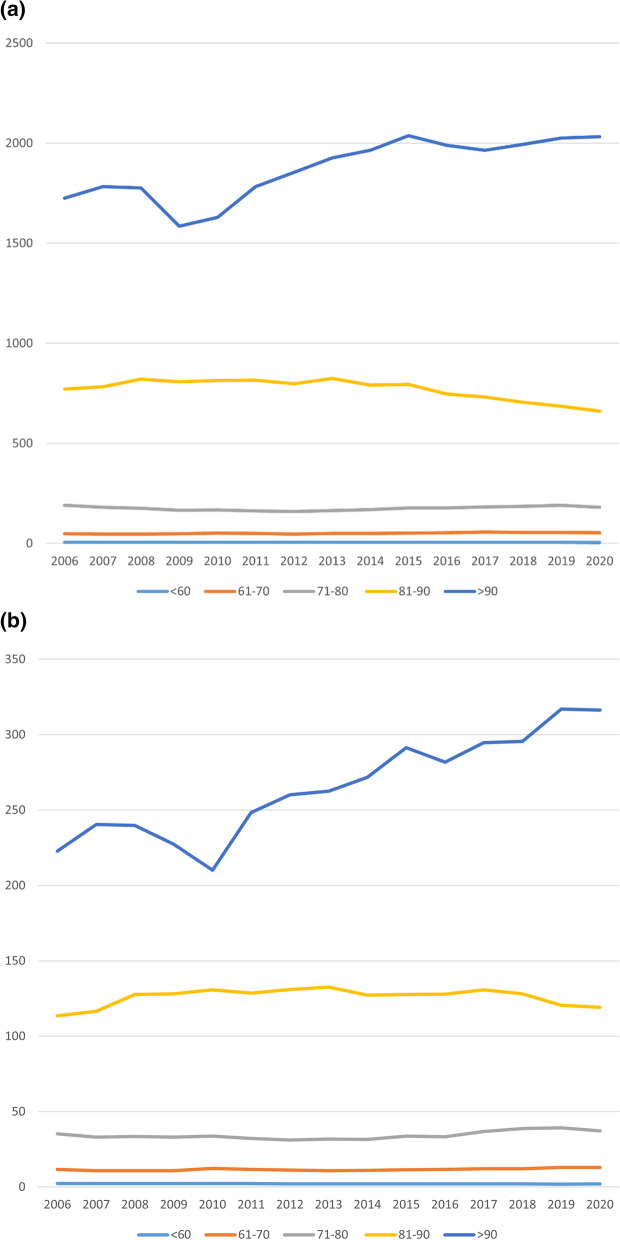
**a** Incidence development of pertrochanteric fractures. **b** Incidence development of subtrochanteric fractures

A representation of the overall incidence development during the study period, divided by age, can be found in Fig. 1a for pertrochanteric and Fig. 1b for subtrochanteric fractures. Both show a sideward trend in those under the age of 90 but an increase of incidence in those over the age of 90.

The increase in incidence in the age groups of 90 and above could be shown to significantly correlate with the year of report in both fracture types with an adjusted *R*^2^ = 0.690 and *F*(1,13) = 32.2 (*p* < 0.001) for pertrochanteric fractures and an adjusted *R*^2^ = 0.865 and *F*(1,13) = 226 (*p* < 0.001) for subtrochanteric fractures, both leading to the assumption of an approximately linear increase in fracture incidence in these age groups with respective correlation coefficients of *B* = 28.2 pertrochanteric fractures per 100,000 and *B* = 7.05 subtrochanteric fractures per 100,000. It should be noted that both analyses showed deviations in QQ plot analysis of the standardised residuals, which may reduce the significance of the reported results by some margin. A significant dependence on sex of these developments could not be shown, suggesting equal prevalence of the development in males and females.

**Fig. 2:**
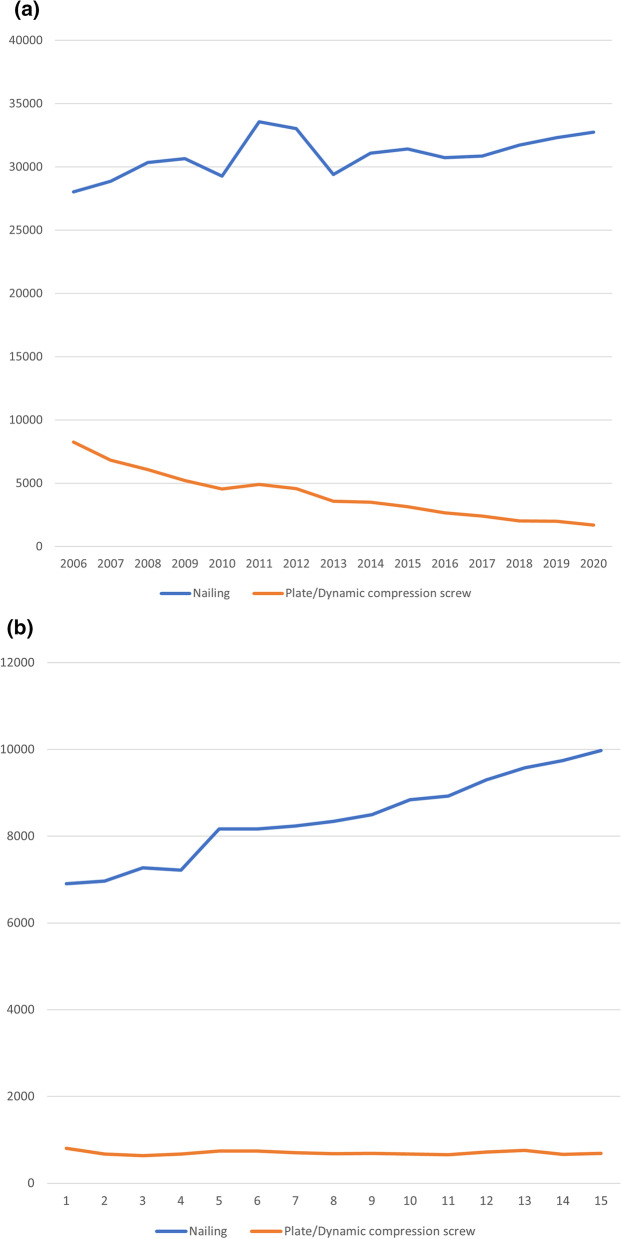
**a** Procedures performed on pertrochanteric fractures. **b** Procedures performed on subtrochanteric fractures

A graphical representation of the prevalence of nailing and plating/compression screw treatments can be seen in Fig. 2a for pertrochanteric fractures and Fig. 2b for subtrochanteric fractures.

A clear trend of increasing use in IMN is apparent, while plates and compression screws are being used less frequently.

Throughout the study period, nails were used in 47.33% ± 3.00% of reported pertrochanteric cases and in 70.51% ± 2.00% of reported subtrochanteric cases. Both shares are showing a sideward trend over the study period. On the other hand, plates and dynamic compression screws were used in 6.52% ± 3.00% of reported pertrochanteric cases and in 15.56% ± 5.00% of reported subtrochanteric cases, while showing a clear downward trend.

**Fig. 3:**
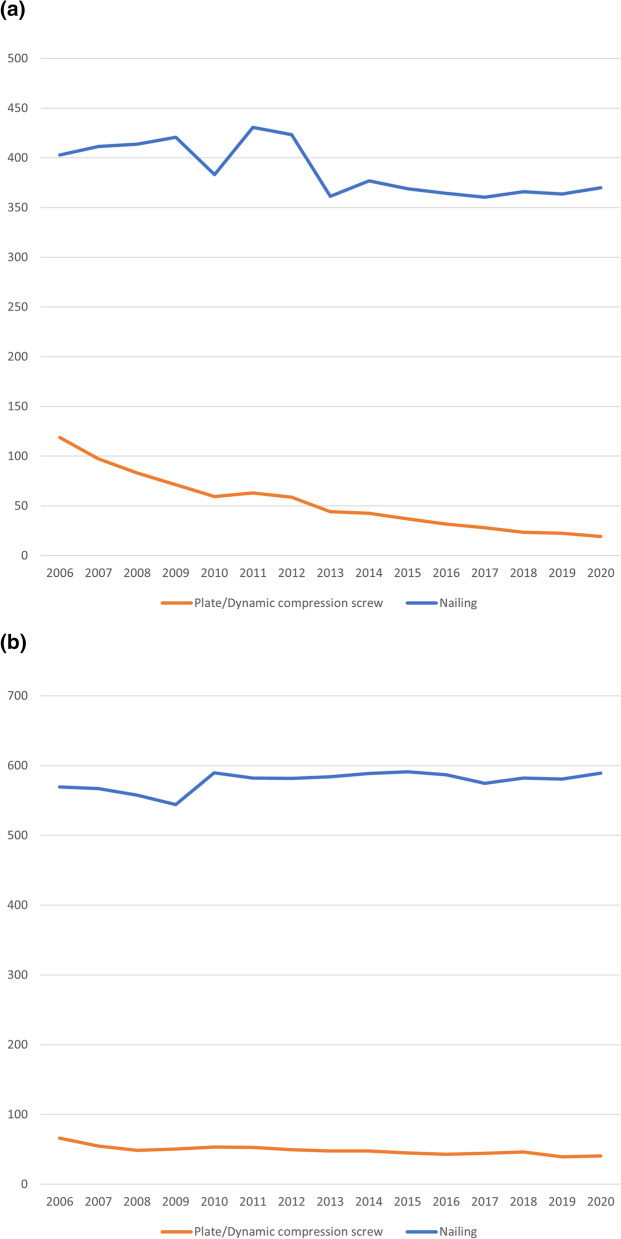
**a** Standardised procedures prevalence on pertrochanteric fractures. **b** Standardised procedures prevalence on subtrochanteric fractures

To combat confounding factors like population size and differing risks of fracture over time, while comparing the trend of the different surgical treatment options, we adjusted all procedures for incidence by dividing the number of procedures performed by their respective annual incidence before analysis. The results are presented in Fig. 3a for pertrochanteric fractures and Fig. 3b for subtrochanteric fractures.

While overall utilisation of nailing techniques has increased over time, the incidence adjusted plots show a sideward trend. Plate and dynamic compression screws, on the other hand, are still decreasing in frequency over the analysed period.

The decrease in plating and compression screw techniques could again be shown to significantly correlate with the year of report in both fracture types with an adjusted *R*^2^ = 0.911 and *F*(1,13) = 145 (*p* < 0.001) for pertrochanteric fractures and an adjusted *R*^2^ = 0.727 and *F*(1,13) = 38.3 (*p* < 0.001) for subtrochanteric fractures, both leading to the assumption of an approximately linear decrease in plating/dynamic compression screw utilisation in these age groups with respective correlation coefficients of *B* = − 0.145 plates/compression screws per diagnoses per 100,000 and *B* = − 0.583 plates/compression screws per diagnoses per 100,000.

**Fig. 4:**
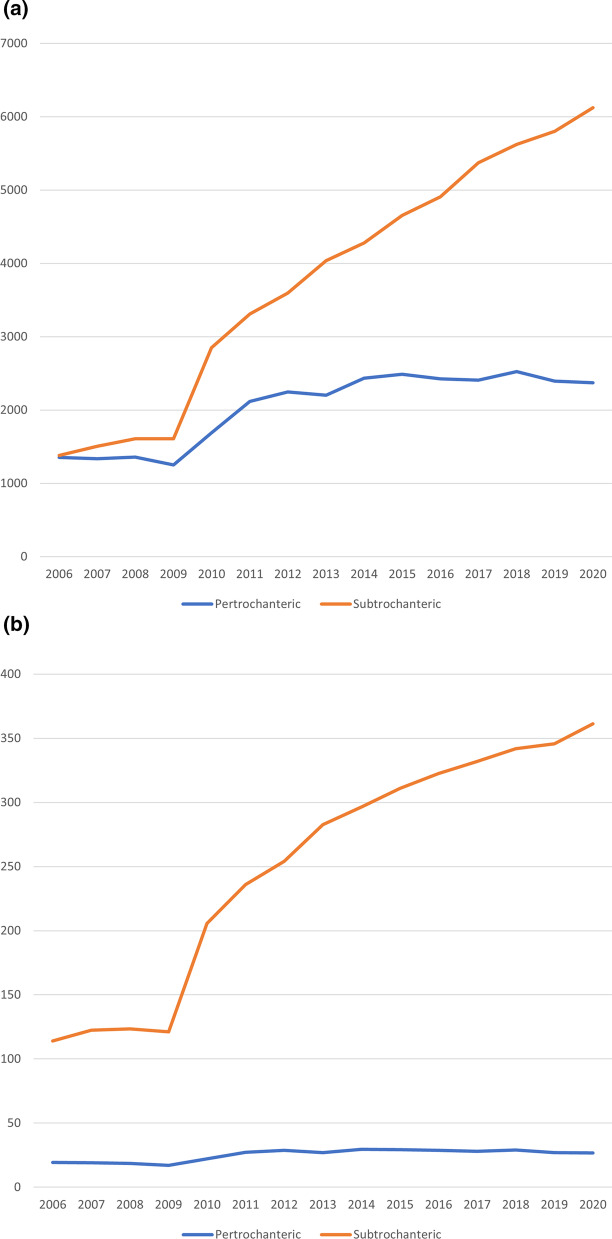
**a** Prevalence of cerclages in both fracture type. **b** Standardised prevalence of cerclages in both fracture types

Augmentative cerclages were also subject to analysis and are represented in Fig. 4a as overall prevalence and Fig. 4b as standardised prevalence for both fracture types.

Overall, the relevance of augmentative cerclages has increased drastically over the study period. While only a total of 2736 cerclages were reported in 2006 for both fracture types, 8493 were reported in 2020. This increase of roughly 210% is mainly due to its increased usage in subtrochanteric fractures, with a distinct bend in standardised prevalence from 2009 to 2010. Even though this bend is present, in analysing the standardised prevalence of cerclages in subtrochanteric fractures with linear regression, we calculated an adjusted *R*^2^ = 0.923 and F (1,13) = 169 (*p* < 0.001) and a correlation coefficient of *B* = 0.047 cerclages per diagnoses per 100,000. This leads to the assumption of approximate linear growth in relevance of cerclages during the study period.

## Discussion

We analysed the incidence and its development in Germany from 2006 to 2020 and detected a mean annualised incidence of 80.08 ± 6.34 pertrochanteric fractures per 100,000 and 14.53 ± 1.50 subtrochanteric fractures per 100,000. Comparable data from the USA regarding subtrochanteric fractures reports an incidence of approximately 15–20 per 100,000 which is comparable to our findings [33]. Comparable incidence rates for pertrochanteric fractures have been reported by Rupp et al. at 108.7 per 100,000 inhabitants in Germany [34]. Data are also available from the USA for those above the age of 65 and are again comparable with an overall incidence of 171 per 100,000 citizens [35].

The sex-dependence of overall mean incidence, with roughly two women to one man affected, was in line with other studies published from different industrial nations with similar healthcare systems like Sweden, the United Kingdom, and Spain [4, 36, 37].

We also found a distinct dependence on age regarding incidence. While in younger age groups (< 60 years old), males were more likely to suffer from a fracture, in older age groups, females were much more likely to suffer from a fracture. A significant development of this relation during the study period could not be found, implying an ongoing and intrinsic discrepancy between the sexes regarding trochanteric and subtrochanteric fracture risk. Influencing factors of incidence seem to affect both sexes in a similar manner. Most of the described age and sex-dependence of fracture incidence is most likely caused by previously described effects of osteoporosis and its associated risk factors of increased age and female sex [38–40]. A study performed by Orwoll et al. in 2006 also links lower androgen concentration to higher fall risks, which also indicates an additional reason for sex-dependence of hip fractures in the elderly, as it could be shown that they are predominantly fall-related [41, 42]. Smoking was also proven as a risk factor for hip fractures by Xu et al. in 2022, and was reported as more prevalent among the male population than among the female population in Germany [43, 44]. Its effect on bone density, even early on in life, could partly explain the higher prevalence among younger males when compared to females, as other risk factors like low oestrogen levels are not yet playing a key role in those groups [45].

Additionally, we found a very prevalent increase in both fracture incidences among the age groups of 90 and older. This increase in incidence, the distinct dependence of incidence on age in both fracture entities, and the ongoing demographic shift to an aging population present alarming signals to the healthcare system.

Especially the increase in incidence of fractures in the very old population leads to the need for better preventative care and the continued implementation of therapeutic concepts for osteoporosis that have been proven to effectively reduce fracture risks to decrease the individual and socioeconomic burden of these cases [46].

Part of this socioeconomical burden associated with the analysed fracture types can be quantified by estimating the direct individual mean treatment cost. A study by Weyler and Gandjour estimated the treatment cost of hip fractures in the first year of treatment at about 19,878€ [47]. This is, of course, highly dependent on individual treatment strategies, but can be used to estimate overall cost. With a summarised incidence of 94.61 fractures per 100,000 and a population of about 83.13 million, this accumulates to approximately 1.563 billion € per year for Germany.

The most dominant treatment option among our cohort was IMN and is further increasing in relevance through the years. This is a welcomed observation, as different studies show favourable clinical outcomes and lower revision rates of IMN when compared to alternate methods, like the sliding hip screw, in both fracture types [48–51]. Also, a cost-analysis of IMN from 2014 further justifies the increased usage of IMN systems as a more cost-effective treatment option in subtrochanteric type A3 fractures than comparative systems, while in the majority of A2 fractures a sliding hip screw remains the more cost-effective option [52].

The observed increase in augmentative cerclages can also be described as preferable, as the literature suggests increased stability and better clinical outcomes in patients that need open fracture reduction, especially when combined with IMN [53, 54]. It is also possible that an increase in fracture severity resulted in the more frequent application of cerclages.

It is likely that more patients with pertrochanteric fractures received plates, DHS or nails but have been misreported or not have been reported at all by the respective hospitals even though they are required to do so by law. This may have resulted in nonreliable data concerning the absolute number of implants used.

Concerning our results and regarding the immense cost of the analysed hip fractures, possible cost reduction needs to be addressed. To reduce healthcare costs and to benefit the victims of hip fractures pre-emptively, preventive measures need to be taken. As we addressed earlier, hip fractures are widely considered to be predominantly caused by falls [8]. Systematic reviews regarding fall risk and potential preventive measures have already shown that regular exercise may significantly reduce it [28, 55]. In addition, other authors suggest that a nutritional assessment to prevent malnutrition may also result in a reduction of falls among the elderly [56]. Another potential factor can be found among medications. Non-selective beta blockers, for example, are associated with an increased fall risk [57]. A medication review and adaption based on individual factors may aid in further reducing the risk of falling [58]. The withdrawal of such fall risk-increasing drugs was even shown to be cost-effective among the elderly in a 2008 study from the Netherlands, resulting in a net cost saving of approximately 1,691€ per patient at the time [59]. Vision impairment, and thus regular vision screenings, may also help to reduce fall risk, as poor vision was previously determined to be an independent risk factor for falls [58, 60]. Additionally, home visit programs to identify fall hazards and to develop behavioural strategies at home could be deployed with promising effect [61].

It needs to be stated that it was previously postulated that a significant amount of hip fractures may, in fact, not be caused by a fall but by other factors like muscle tension and movement [62, 63]. This leads to the conclusion that preventive measures not only need to reduce fall risks but also need to address factors increasing the risk of spontaneous fractures. While osteoporosis and associated loss of bone density is one major factor, other factors like the treatment of osteoporosis and malnutrition may need to be addressed, as well [20, 23]. Namely, there is a proven association between the intake of bisphosphonate therapy and femur fractures [64, 65]. The increased utilisation of alternative treatments, like biologicals such as denosumab, may be able to reduce the associated risk of stress-induced atypical fractures, though there are some speculations that the similarity in mechanisms of action may be too similar so that an associated risk may prevail while treatment costs are now higher than with traditional approaches [66].

While this study provides a trustworthy assessment of incidence and treatment modalities of per- and subtrochanteric fractures, it needs to be pointed out that its design bears several limitations. For one, the underlying database does not provide any additional information about individual patient history or outcome. This means that we are not able to assess the observed developments reliably regarding their impact. We can only assume the developmental implications by supplementing our findings with other research. The study’s design, however, is perfectly able to be reproduced in the future to assess the real-world impact of preventive measures or large-scale events like the Covid-19 pandemic. Already it has been shown to have a distinct impact on the occurrence of traumatic injuries [67]. In the future, this can be used to better assess the impact on the healthcare system on a nationwide scale.

## Conclusions

We provided incidence data on per- and subtrochanteric fractures and their treatment. We found an ongoing increase of incidence among the elderly and an increase in intramedullary nailing. Additionally, we found an ongoing increase in augmentative cerclages.

Not only could we show an age-dependence of the incidences, but also a sex-dependence that seems to remain consistent in its development. While younger males (< 60 years old) are at a higher risk than their female peers, older females are at significantly higher risk than their male peers. We explored potential explanations for this observation, like osteoporosis and its associated risk factors.

We also explored the economic implications of our findings and potential preventive measures, as well as their feasibility in a modern health economy.

## Data Availability

The datasets generated and/or analysed during the current study are available from the corresponding author upon reasonable request. Raw data concerning the German hospital statistics are also available upon request directly from the Federal Bureau of Statistics or partly from its health-reports website (gbe-bund.de).

## Abbreviations

BfArM: German Federal Institute for Drugs and Medical Devices
DHS: Dynamic hip screw
ICD-10 GM: International Statistical Classification of Diseases and Related Health Problems 10th revision German Modified
IMN: Intramedullary nail
OPS: Operationen- und Prozedurenschlüssel
WHO ICPM: World Health Organization International Classification of Procedures in Medicine
